# Supplementary figures and images for: Mitochondrial metabolism is rapidly re-activated in mature neutrophils to support stimulation-induced response
Source: Front Immunol. 2025 Apr 28;16:1572927. doi: 10.3389/fimmu.2025.1572927 (PMC12066771; doi:10.3389/fimmu.2025.1572927)

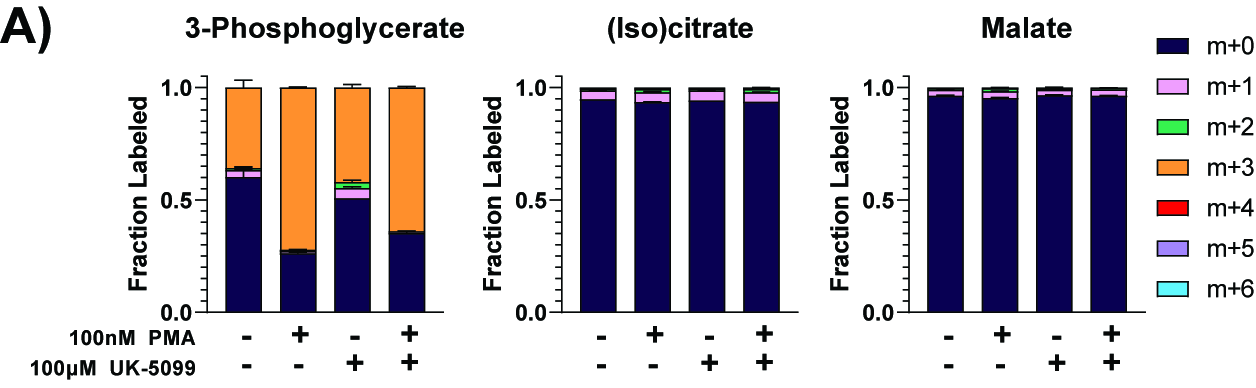

Supplement: Supplementary Figure 1 — PMA does not increase TCA cycle labeling. U13C-glucose labeling into glycolysis and TCA intermediates in primary human neutrophils treated under specified conditions for 1 hour. [file Image1.tif]

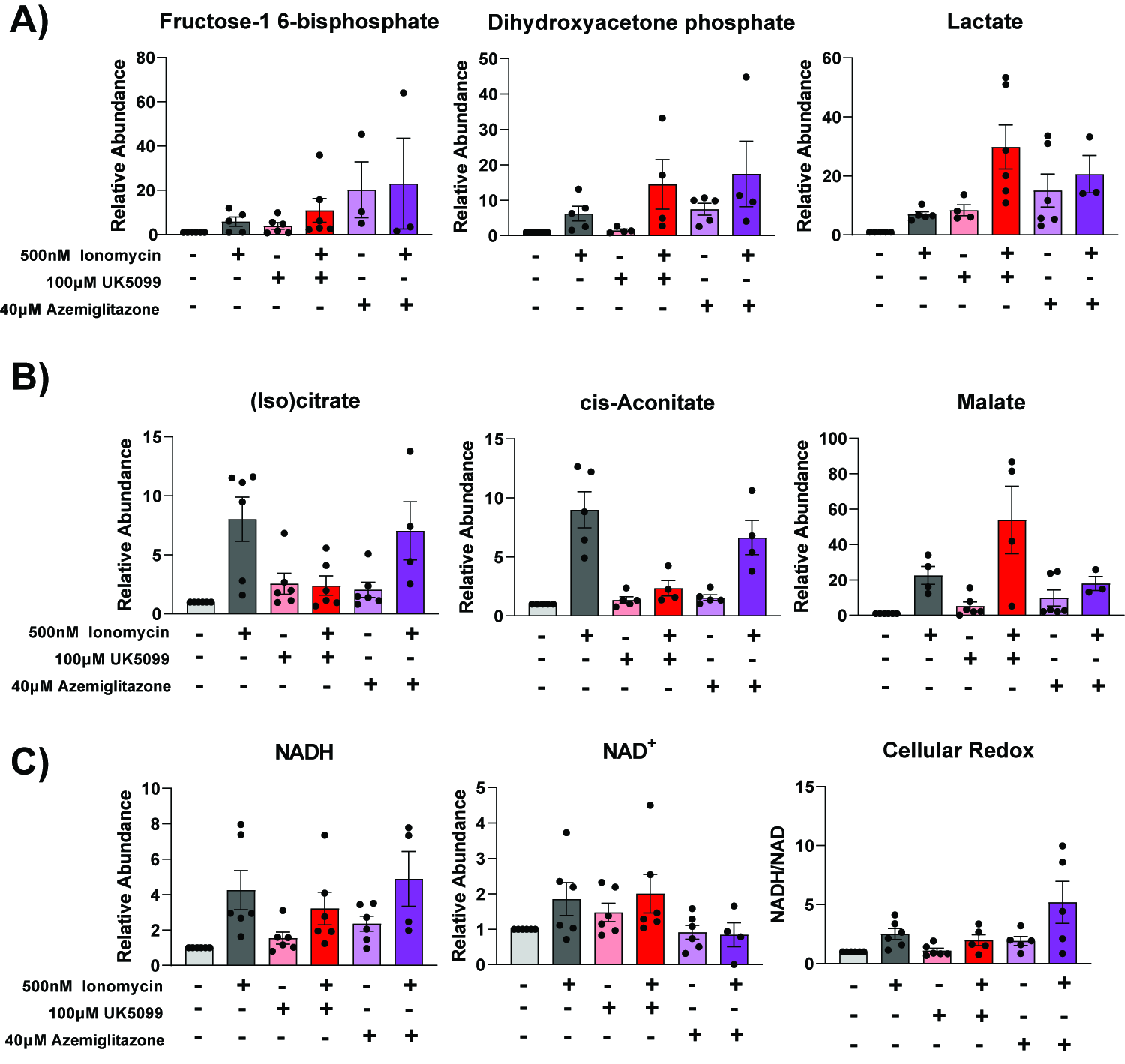

Supplement: Supplementary Figure 2 — Changes in metabolite abundance in human peripheral blood neutrophils caused by Ionomycin stimulation and MPC inhibition. Relative abundance of (A) glycolysis intermediates, (B) TCA intermediates, and (C) redox metabolites from 6 different donors was compiled and normalized by protein content and made relative to the unstimulated condition from the respective donor. Cells were pretreated with MPC inhibitors for 45 minutes and then stimulated for 1 hour. [file Image2.tif]

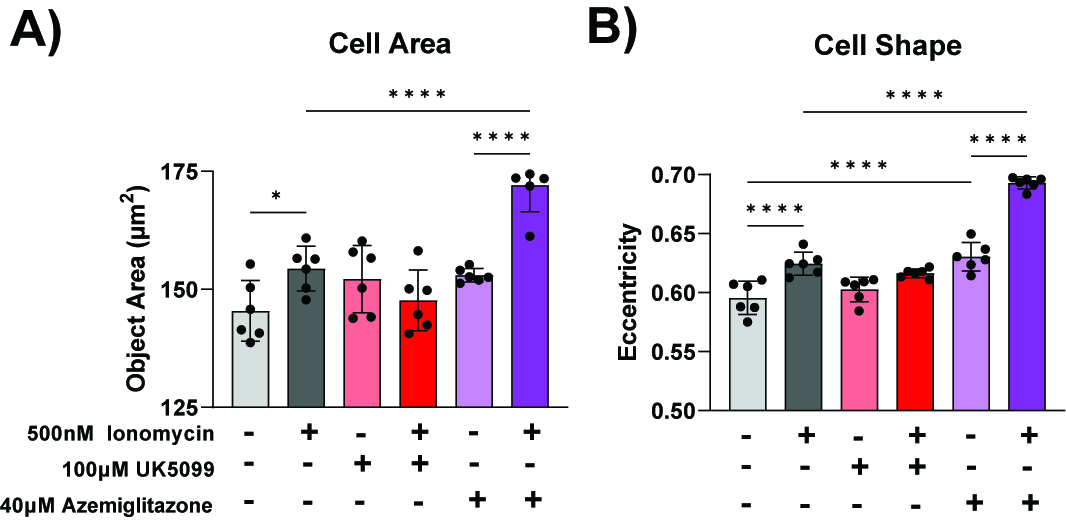

Supplement: Supplementary Figure 3 — Effects of ionomycin stimulation and MPC inhibition on primary human neutrophil morphology. (A) Cell size and (B) cell eccentricity measurements in neutrophils treated under specified conditions for 4 hours. Significance was determined by ordinary one-way ANOVA and Šídák’s multiple comparisons test. [file Image3.tif]

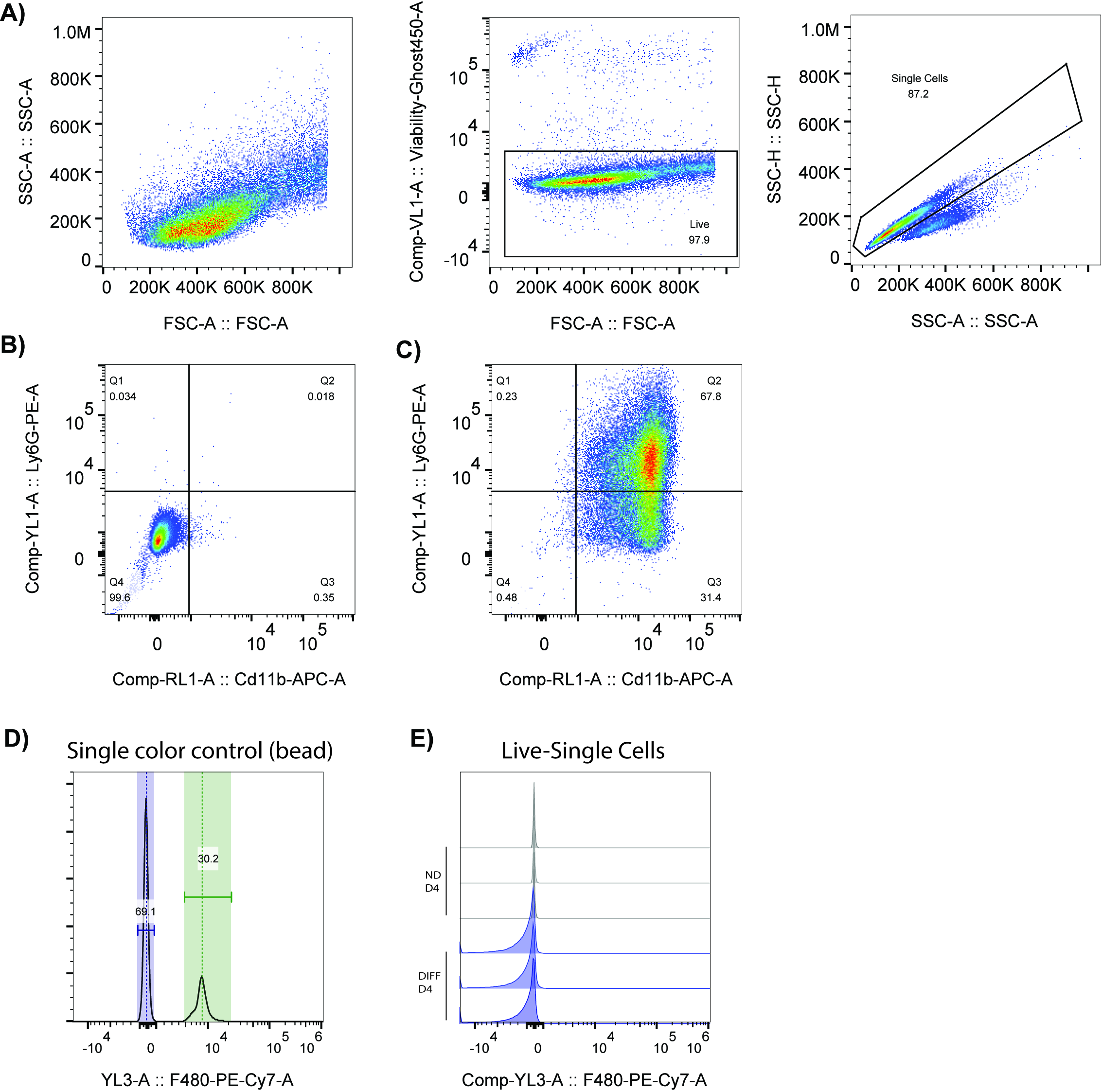

Supplement: Supplementary Figure 4 — Flow cytometry gating scheme of ER-Hoxb8 neutrophil differentiation. (A) Gating strategy to get live single cells. Gating strategy for CD11b and Gr-1 in (B) undifferentiated and (C) day-4 differentiated ER-Hoxb8 neutrophils. Expression of F4/80 in (D) single color bead control vs (E) undifferentiated and day-4 differentiated ER-Hoxb8 cells. [file Image4.tif]
